# Influences of different sedatives on gastric antrum contraction in patients with acute brain injury

**DOI:** 10.3389/fneur.2024.1492604

**Published:** 2024-12-20

**Authors:** Meihua Mei, Mingli Yao, Jingchao Li, Chunfang Qiu, Yufang Wang, Yan Li, Lei Shi, Lingyan Wang, Bin Ouyang

**Affiliations:** Neurosurgery ICU, The First Affiliated Hospital, Sun Yat-sen University, Guangzhou, China

**Keywords:** acute brain injury, sedatives, ultrasonography, gastric motility, propofol, midazolam, dexmedetomidine

## Abstract

**Background:**

Patients with acute brain injury (ABI) often exhibit gastrointestinal motility disorder and the administration of sedatives may exacerbate the gastrointestinal dysfunction. This study aims to evaluate the influences of different sedatives on gastric antrum contraction in patients with acute brain injury (ABI).

**Methods:**

A prospective observational study was performed in 37 adult ICU patients with ABI, and 18 adult healthy volunteers were recruited as normal controls. Gastric motility, including frequency (ACF), amplitude (ACA), and motility index (MI), was measured with ultrasound before and after using sedatives, either propofol (Group A), midazolam (Group B), or dexmedetomidine (Group C). The influences of different sedatives on gastric motility were analyzed.

**Results:**

All patients with acute brain injury (*n* = 37) exhibited decreased ACF and MI compared with those in healthy control (*n* = 18) (ACF: 2.41 ± 0.89 times/2 min in ABI vs. 4.5 ± 0.39 times/2 min in control, MI: 1.25 ± 0.57 in ABI vs. 3.59 ± 0.24 in control, *p* = 0.001). All sedatives, either propofol, midazolam, or dexmedetomidine, had inhibited effects on gastric motilities [In Group A (*n* = 13), 1.14(0.59, 1.44) before vs. 0.84(0.09, 0.83) after, *p* = 0.002; In group B (*n* = 12), 1.48(0.73, 1.62) before vs. 0.31(0.04, 0.58) after, *p* = 0.007; In Group C (*n* = 12), 2.74(1.70, 3.01) before vs. 1.39(0.70, 2.28)]. However, dexmedetomidine showed significantly less inhibition either on ACA or MI compared with propofol and midazolam (ACA 20.67 ± 33.59% in dexmedetomidine, 51.50 ± 32.83% in propofol, 60.43 ± 22.40% in midazolam, *p* = 0.002; MI 36.00 ± 34.77% in dexmedetomidine, 60.69 ± 27.49% in propofol, 68.81 ± 20.84% in midazolam, *p* = 0.012).

**Conclusion:**

Patients with ABI exhibited decreased gastric motility. All sedatives, either propofol, midazolam, or dexmedetomidine, had inhibited effects on gastric motilities. Dexmedetomidine has less inhibitory effects on ACA and MI compared with propofol and midazolam.

## Introduction

Patients with acute brain injury (ABI) exhibit gastrointestinal motility disorder (1, 2). The autonomic nerve dysfunction resulting from ABI results in gastrointestinal muscle dysmotility, and the severity is associated with the intracranial pressure (3). On the other hand, ABI can activate the hypothalamic–pituitary–adrenal (HPA) axis and induce a systemic stress response that triggers intestinal smooth muscle inflammation, further leading to the disorder of intestinal smooth muscle contraction (4).

In patients with ABI, sedatives were indicated for controlling anxiety, pain, discomfort, agitation, facilitating mechanical ventilation, and also for “neuro-specific” indications such as reducing cerebral metabolic demands, and enhancing cerebral tolerance to ischemia (5). Sedatives are indispensable therapeutic components in therapeutic measures such as reducing intracranial pressure, maintaining temperature, and controlling seizure (5). However, the effects of sedation on gastric motility in patients with ABI have rarely been well studied.

Gastric antrum ultrasound is an advanced technique developed in recent years (6, 7). Antrum contraction can be observed to evaluate gastric motility directly. It is non-invasive and can be performed at the bedside. In this study, we used gastric antrum ultrasound to assess the effects of different sedatives on gastric motility in patients with ABI and compare their degree of inhibition of gastric antral contractions.

## Materials and methods

### Study design

A prospective observed study was conducted. The sedative, either propofol (Group A), midazolam (Group B), or dexmedetomidine (Group C), was used according to the patients’s needs, and light sedation was applied. Gastric motility was measured with gastric antrum ultrasound before and after the administration of sedatives. The effects of different sedatives on gastric antrum contraction, which includes frequency (ACF), amplitude (ACA), and motility index (MI), were analyzed and compared. In addition, 18 adult healthy volunteers were recruited as normal controls to compare with patients with ABI.

This research was approved by the Ethics Committee of Clinical Research and Experimental Animals of the First Affiliated Hospital of Sun Yat-sen University (Ethics No. [2018] 161). The institutional review board waived the requirement for informed consent since no intervention was performed and no personally identifiable information appeared.

### Patients recruitment

**Figure 1:**
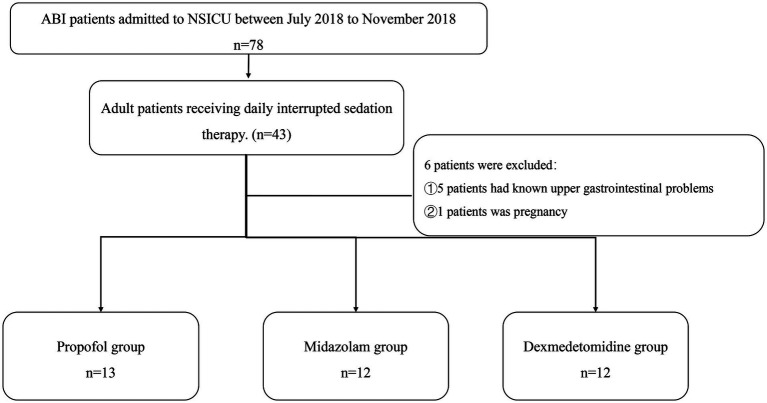
Flowchart. NSICU, neurosurgery intensive care unit; ABI, acute brain injury.

Patients hospitalized in the neurosurgery ICU of the First Affiliated Hospital, Sun Yat-sen University, from July 2018 to November 2018 were recruited (Figure 1).

Inclusion criteria: 18–80 years old. Patients underwent ABI including intracranial hemorrhage, post-selective operation with brain tumors, or traumatic brain injury. Need therapy of short-acting sedatives like midazolam, propofol, or dexmedetomidine, and no oral hypnotic drugs were used.

Exclusion criteria: known upper gastrointestinal anatomical problems. Pregnancy.

### Sedation protocol

The Richmond Agitation-Sedation Scale (RASS) was maintained at −2 ~ 1 points, meaning the depth of sedation was maintained at light sedation. A daily wake-up procedure was performed on each patient at 6–9 a.m.

### Gastric antrum ultrasound

Gastric antrum ultrasound imaging was performed before and 20 min after sedation. All ultrasonic gastric motility monitoring in this study was completed between 9 and 10 a.m., which is 2 h later after the daily wake-up procedure. Enteral nutrition was performed at 60–80 mL/h through a nasogastric tube. Gastric antrum images were obtained with a 2.5–6 MHz curvilinear probe (SONIMAGE HS1 portable ultrasonic, Konica Minolta). Ultrasound examination was performed at 30-degree head-of-bed elevation and supine position. The antrum was located after identifying the liver’s left anterior lobe, the pancreas’s head, and the abdominal aorta. ACF was measured for 6 min, and an average number of gastric antrum contractions were observed every 2 min. The maximum gastric antral diastolic area (S_max_) and minimum contraction area (S_min_) were measured thrice. ACA was calculated as follows: ACA = (S_max_ - S_min_)/ S_max_. MI was calculated as follows: MI = ACF × ACA. The same experienced sonographer performed all of the scans, as the force of ultrasound probe placement may affect the interpretation of the cross-section.

### Bowel sounds auscultation

Bowel sound auscultation was conducted before and 20 min after sedation. Bowel sound auscultation was for 3 min and the frequency of bowel sounds was calculated per minute.

### Other clinical data collection

The following clinical data were collected: age, gender, disease diagnosis, complications such as diabetes and coronary heart disease, body mass index (BMI), GCS score, and the utilization of basic medications such as proton pump inhibitors, or nonsteroidal anti-inflammatory drugs, or opioids, enrollment time after admission to ICU, length of ICU stay, and death.

### Statistical analysis

The SPSS software (version 23.0) was used for the statistical tests. Normally distributed data were expressed as mean ± standard deviation, non-normal distribution data median (interquartile ranges, IQR). The paired student’s test or the paired rank-sum test was adopted to analyze the difference before and after the sedative therapy. The least significant difference *t*-test (LSD-t) or Bonferroni method was applied for multiple comparisons. *p* < 0.05 was considered to be statistically significant.

## Results

### Baseline characteristics

This study included 37 patients. Among them, 30 cases were with intracranial hemorrhage, 5 cases were with brain tumor, and 2 cases were with traumatic brain injury. In addition, 18 healthy volunteers were recruited for this study to compare gastric motility in patients with ABI. The sedative dosage was recorded. In this study, the dosage of propofol (*n* = 13) was 0.036–0.043 mg/kg/h, the dosage of midazolam (*n* = 12) was 0.031–0.067 mg/kg/h, and the dosage of dexmedetomidine (*n* = 12) was 0.171–0.230 mg/kg/h. It is shown in Table 1.

**Table 1:** Characteristics of patients.

Group	Propofol (*n* = 13)	Midazolam (*n* = 12)	Dexmedetomidine (*n* = 12)	Health volunteers (*n* = 18)	*p*-value
Age (years)	54.6 ± 16.2	57.3 ± 12.3	55.7 ± 14.7	32.6 ± 2.1	0.47
Genger (male), No. (%)	6 (46.2)	7 (58.3)	6 (50)	7 (38.9)	0.39
BMI (kg/m^2^)	21.5 ± 1.5	21.25 ± 2.1	20.7 ± 1.9	21.2 ± 1.1	0.26
GCS score	7.8 ± 1	8.2 ± 0.8	7.5 ± 0.3		0.38
Diagnosis, No. (%)					
ICH	12 (92.3)	7 (58.3)	11 (91.7)	NA	
TBI	0	2 (16.7)	0	NA	
Brain tumor	1 (7.7)	3 (25.0)	1 (8.3)	NA	
Complications, No. (%)					
Type 2 diabetes mellitus	3 (23.1)	2 (16.7)	3 (25.0)	0	0.37
Coronary Heart Disease	0	0	0	0	
Sedatives					
Dosage (mg/kg/h)	0.036–0.043	0.031–0.067	0.171–0.230	NA	
RASS score	−1 ± 0.5	−1.3 ± 0.4	−1.2 ± 0.3	NA	0.48
Basic medications, No. (%)					
Proton pump inhibitors	13 (100.0)	12 (100.0)	12 (100.0)	NA	1
Nonsteroidal anti-inflammatory drugs	4 (30.8)	3 (25.0)	4 (33.3)	NA	0.43
Opioids	9 (69.2)	9 (75.0)	8 (66.7)	NA	0.44
Enrollment time after admission to ICU, No. (%)					
5	4 (30.8)	3 (25.0)	5 (41.7)	NA	0.41
6	5 (38.4)	5 (41.7)	4 (33.3)	NA	0.52
7	4 (30.8)	4 (33.3)	3 (25.0)	NA	0.31
Length of ICU stay, median days (range)	12 (7–41)	10 (7–37)	13 (6–31)	NA	0.29
Number of deaths, No. (%)	2 (15.4)	2 (16.7)	3 (25.0)	NA	0.33

### Gastric motility in patients with ABI

In this study, patients with ABI showed lower ACF (2.41 ± 0.89 times/2 min) and MI (1.25 ± 0.57) than health volunteers (ACF: 4.5 ± 0.39 times/2 min, *p* = 0.001; MI: 3.59 ± 0.24, *p* = 0.001). There was no significant inhibition of ACA (50.80 ± 11.60%), as shown in Table 2.

**Table 2:** The status of gastric motility.

Main outcomes	Patients with ACI	Health volunteers	*t* value	*p*-value
ACF (times/2 min)	2.41 ± 0.89	4.5 ± 0.39	15.23	0.001
ACA (%)	50.80 ± 11.60	55 ± 12	0.54	0.21
MI	1.25 ± 0.57	3.59 ± 0.24	11.57	0.001

ACI, acute craniocerebral injury; NA, not available.

### Influences of propofol on gastric antrum contraction and bowel sounds

In patients receiving propofol (*n* = 13), the ACA decreased significantly (53.31 ± 9.76% before vs. 26.41 ± 18.01% after, *p* = 0.002), ACF decreased significantly (2.27 ± 0.78 times/2 min before vs. 1.08 ± 0.74 times/2 min after, *p* = 0.002), and MI decreased significantly (1.14(0.59, 1.44) before vs. 0.84(0.09, 0.83) after, *p* = 0.002). The bowel sounds had no significant change after using propofol (3 (2.5, 3) times/min before vs. 3 (2, 3) times/min after, *p* = 0.317). It is shown in Table 3.

**Table 3:** Effects of propofol on gastric antrum contraction and bowel sounds.

Main outcomes	Before intervention	After intervention	D-value	Inhibitory ratio (%)	*t* value	*p*-value
ACF (times/2 min)	2.27 ± 0.78	1.19 ± 1.05	−1.08 ± 0.74	51.50 ± 32.83	5.56	0.003
ACA (%)	53.31 ± 9.76	26.41 ± 18.01	−26.90 ± 16.77	49.69 ± 33.48	8.72	0.002
MI	1.14 (0.59, 1.44)	0.84 (0.09, 0.83)	−0.40 ± 0.59	60.69 ± 27.49	7.45	0.002
Bowel sounds (times/min)	3 (2.5, 3)	3 (2, 3)	0.23 (−0.27, 0.73)	NA		0.317

NA, not available.

### Influences of midazolam on the contraction of the gastric antrum and bowel sounds

In patients receiving midazolam (*n* = 12), the ACA decreased significantly (53.35 ± 13.87% before vs. 21.04 ± 12.47% after, *p* = 0.002), ACF decreased significantly (2.23 ± 1.04 times/2 min before vs. 1.32 ± 1.04 times/2 min after, *p* = 0.007), MI decreased significantly (1.48 (0.73, 1.62) before vs. 0.31 (0.04, 0.58) after, *p* = 0.007). The bowel sounds decreased after using midazolam (3 (2.25, 3) before vs. 2 (2, 3) after, *p* = 0.034). It is shown in Table 4.

**Table 4:** Effect of midazolam on gastric antrum contraction and bowel sounds.

Main outcomes	Before intervention	After intervention	D-value	Inhibitory ratio (%)	*t* value or *Ζ*	*p*-value
ACF (times/2 min)	2.23 ± 1.04	1.32 ± 1.04	−0.96 ± 1.00	60.43 ± 22.40	5.78	0.007
ACA (%)	53.35 ± 13.87	21.04 ± 12.47	−32.31 ± 14.03	37.83 ± 33.75	9.63	0.002
MI	1.48 (0.73, 1.62)	0.31(0.04, 0.58)	−0.89 ± 0.51	68.81 ± 20.84	10.72	0.002
Bowel sounds (times/min)	3 (2.25, 3)	2 (2, 3)	−0.5 (0, 1)	NA	7.84	0.034

NA, not available.

### Influences of dexmedetomidine on the contraction of the gastric antrum and bowel sounds

In patients receiving dexmedetomidine (*n* = 12), ACF decreased significantly (2.71 ± 0.86 times/2 min before vs. 1.85 ± 0.86 times/2 min after, *p* = 0.01), MI decreased significantly (1.37 (0.85, 1.5) before vs. 0.9 (0.35, 1.14) after, *p* = 0.01), and no significant difference in ACA (45.54 ± 9.98% before vs. 34.44 ± 16.04 after, *p* = 0.099) and bowel sounds (3 (2, 3) before vs. 3 (2.25, 3) after, *p* = 1). It is shown in Table 5.

**Table 5:** Effect of dexmedetomidine on gastrointestinal motility.

Main outcomes	Before intervention	After intervention	D-value	Suppression ratio (%)	*t* value or *Ζ*	*p*-value
ACF (times/2 min)	2.71 ± 0.86	1.85 ± 0.86	−0.86 ± 0.94	20.67 ± 33.59	5.79	0.01
ACA (%)	45.54 ± 9.98	34.44 ± 16.04	−11.10 ± 19.97	29.25 ± 31.36	0.72	0.099
MI	1.37 (0.85, 1.5)	0.7 (0.35, 1.14)	−0.28 ± 0.90	36.00 ± 34.77	6.71	0.01
Bowel sounds (times/min)	3 (2, 3)	3(2.25, 3)	0 (0, 0)	NA	0.62	1

NA, not available.

### Comparison of influences of midazolam, propofol, and dexmedetomidine on gastric antrum contraction

**Figure 2:**
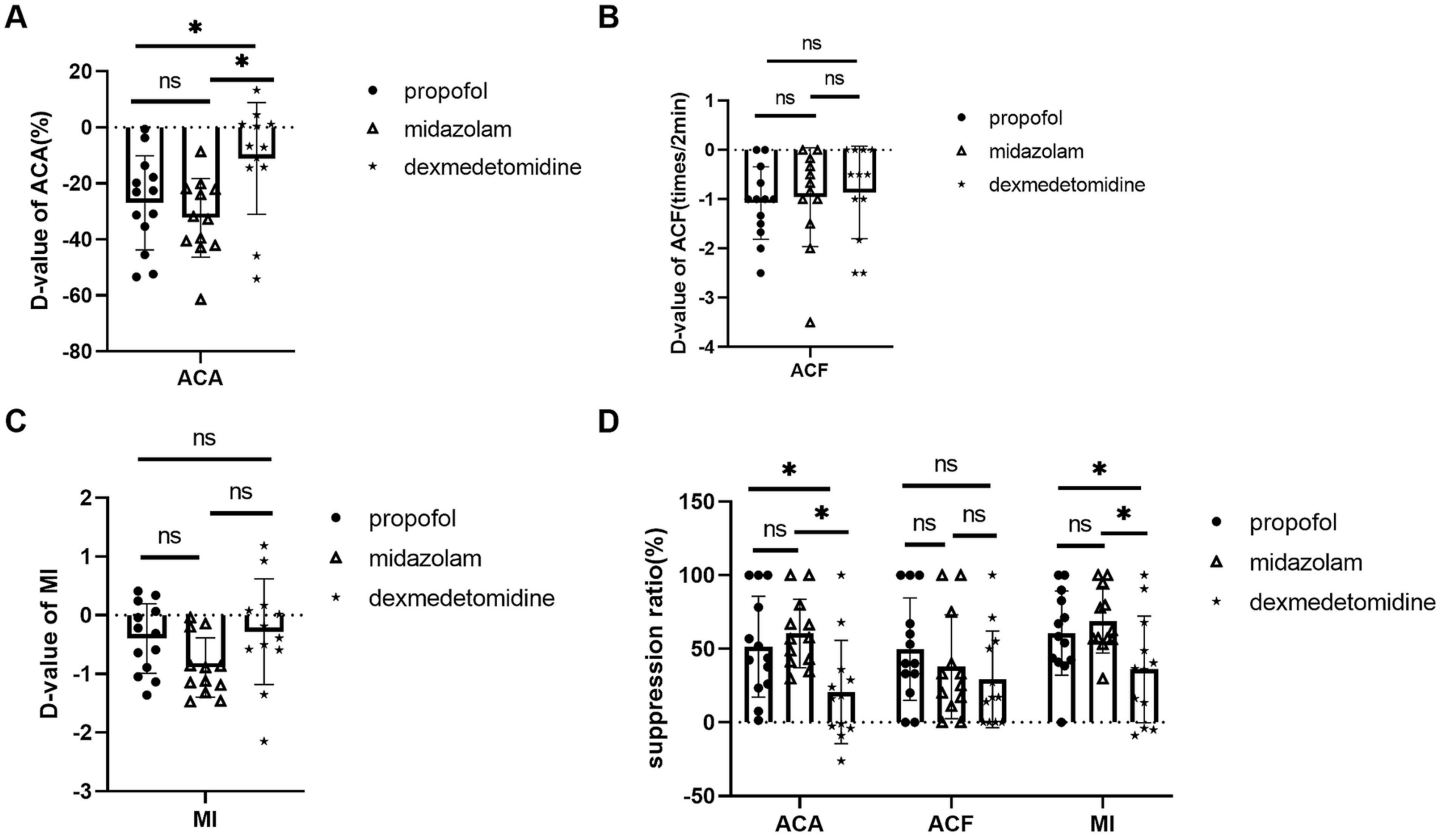
Comparison of midazolam, propofol, and dexmedetomidine effects on ACA, ACF, and MI. **(A)** D-value of ACA of 3 groups. **(B)** D-value of ACF of 3 groups. **(C)** D-value of MI of 3 groups. **(D)** The three groups’ suppression ratios of ACA, ACF, and MI. Ns: no significant difference. *p ≤ 0.05 between groups.

Dexmedetomidine showed less inhibitory effects on ACA compared with midazolam and propofol. D-value of ACA was −11.10 ± 19.96% in dexmedetomidine, −32.21 ± 14.03% in midazolam, −26.90 ± 16.77% in propofol, *p* = 0.003. ACA suppression ratio was 20.67 ± 33.59% in dexmedetomidine, 60.43 ± 22.40% in midazolam, 51.50 ± 32.83% in propofol, *p* = 0.002 (Figures 2A,D).

Dexmedetomidine also showed less inhibitory effects on MI. MI suppression ratio was 36.00 ± 34.77% in dexmedetomidine, 68.81 ± 20.84% in midazolam, 60.69 ± 27.49% in propof ol, *p* = 0.012. D-value of MI was −0.28 ± 0.90 in dexmedetomidine, −0.89 ± 0.51 in midazolam, −0.4 ± 0.59 in propofol, *p* = 0.066 (Figures 2C,D).

There was no statistical difference in ACF. D-value of ACF was −1.08 ± 0.74 times/2 min in propofol, −0.96 ± 1.00 times/2 min in midazolam, 0.86 ± 0.94 times/2 min in dexmedetomidine, *p* = 0.811. ACF suppression ratio 49.74 ± 34.81% in propofol, 37.87 ± 35.24% in midazolam, and 29.0 ± 32.8% in dexmedetomidine, *p* = 0.518 (Figures 2B,D).

## Discussion

The clinical assessment of gastric motility has relied on clinical symptoms such as bloating and vomiting, which are subjective and untimely. Some studies used electrogastrogram to assess gastric motility and gastric emptying (8, 9). However, the electrogastrogram can only represent gastric electrical activity and does not reflect effective gastric contraction. In this study, gastric motility was measured using bedside gastric antrum ultrasound. The gastric antral ultrasound can quantify the contraction amplitude and frequency of the antrum (10). Also, it is noninvasive, easy to operate, and can be performed at the bedside to observe the dynamic effect of clinical treatments. We found that ACF and MI deteriorated in patients with ABI. The finding is consistent with previous studies, which showed gastric motility disorders are severe (11, 12).

### Light sedation has an influence on gastric motility in ABI patients

Sedation is a common treatment for brain injury. Previous studies that explored the effects of sedation on gastric motility in patients with ABI have focused on deep sedation (13, 14). However, light sedation is the current trend in ICU sedation management (5, 15). This study found that light sedation can also reduce gastric motility in patients with ABI.

### Weaker gastric motility inhibitory effect of dexmedetomidine

This study also compared the inhibitory effects of different sedatives on gastric motility. We found dexmedetomidine had less inhibitory effects on gastric motility than propofol and midazolam. Midazolam, a short-acting benzodiazepine, acts directly on the *γ*-aminobutyric ABId receptor (16). Propofol acts on the subtype A of the γ-aminobutyric ABId receptor (17). Different from propofol or midazolam, dexmedetomidine is a highly selective α^2^ agonist, having mild sedative, mild analgesic, and antisympathetic properties (18). Dexmedetomidine showed a less inhibitory effect than midazolam and propofol, possibly due to its lighter sedation effect and sympathoexcitatory depressant function (19). In addition, dexmedetomidine has the ability to attenuate intestinal ischemia–reperfusion injury, inhibit the inflammatory response, and ameliorate the stress response (20). This could also rationalize the weaker inhibition of gastric antral contraction by dexmedetomidine.

### Midazolam caused a decrease in bowel sounds

This study also observed bowel sounds to evaluate intestinal motility. We found that midazolam suppresses bowel sounds, but propofol and dexmedetomidine had no significant inhibition of bowel sounds. However, there are no studies to explore the effects of sedative medications on bowel sounds in patients with ABI. We conjecture that this might be related to the influence of sedatives on gastrointestinal hormones. Narchi et al. (21) found that sedatives, including midazolam and propofol, affect gastrin secretion. Early animal experiments revealed that midazolam intervention could cause a decrease in the secretion of motilin (22, 23). Xu et al. (24) also found that propofol can increase the levels of gastrin and vasoactive intestinal peptide in patients undergoing gastrointestinal endoscopy. Moreover, in a clinical study of open surgery for colon cancer, patients undergoing intravenous anesthesia involving dexmedetomidine had a quicker recovery of gastrointestinal motility, and the levels of prokinetic gastrointestinal hormones such as motilin and gastrin in the plasma increased (25). Thus, It could be explained by the different mechanisms of midazolam, propofol, and dexmedetomidine affecting gastrointestinal motility and bowel sound.

### Limitations and strengths

This is a prospective observational study. There are some limitations in this study. First, the clinical application of basic drugs such as opioids (12), proton pump inhibitors, and non-steroidal anti-inflammatory drugs (26) may influence gastric motility. However, the before-and-after comparison was employed in this study, which mitigated the errors brought by other therapies. Second, in this study, radioactive nuclides, gastrointestinal electrical examination, and capsule endoscopy were not utilized for the detection of gastrointestinal motility because these methods are not suitable for before-and-after comparison of short-acting sedatives. By contrast, gastric antrum ultrasound can be operated non-invasively and enables dynamic re-examination. Nevertheless, further studies were needed to explore the specific mechanisms of different sedatives affecting gastric motility.

## Conclusion

Patients with ABI exhibited decreased gastric motility. All sedatives, either propofol, midazolam, or dexmedetomidine, had inhibited effects on gastric motilities. Dexmedetomidine has less inhibitory effects on ACA and MI compared with propofol and midazolam.

## Data Availability

The original contributions presented in the study are included in the article/supplementary material, further inquiries can be directed to the corresponding author.
